# The effect of almond consumption on elements of endurance exercise performance in trained athletes

**DOI:** 10.1186/1550-2783-11-18

**Published:** 2014-05-11

**Authors:** Muqing Yi, Jinde Fu, Lili Zhou, Hong Gao, Chenguang Fan, Jing Shao, Baohua Xu, Qirong Wang, Juntao Li, Guangwei Huang, Karen Lapsley, Jeffrey B Blumberg, C-Y Oliver Chen

**Affiliations:** 1 Center for Sports Nutrition, National Institute of Sports Medicine, 1st Anding Road, Chaoyang District, Beijing 100029, China; 2 Chinese Baiyi Cycling Team, Fengtai District, Beijing 100072, China; 3 Almond Board of California, Modesto, CA 95354, USA; 4 Antioxidants Research Laboratory, Jean Mayer USDA Human Nutrition Research Center on Aging, Tufts University, Boston, MA, USA

**Keywords:** Almonds, Exercise performance, Substrate oxidation, Antioxidant defense capacity

## Abstract

**Background:**

Almonds are a healthy tree nut food with high nutrient density. Their consumption has been shown to ameliorate oxidative stress, inflammation, etc. The objective of the study was to examine the effect of almonds on elements of endurance exercise performance in trained athletes.

**Methods:**

A 10-week crossover, placebo controlled study was conducted. Eight trained male cyclists and two triathletes were randomly assigned to consume 75 g/d whole almonds (ALM) or isocaloric cookies (COK) with equal subject number. They consumed the assigned food for 4 wks and then the alternate food for another 4 wks. They underwent 3 performance tests including 125-min steady status exercise (SS) and 20-min time trial (TT) on an indoor stationary trainer at the start of the study (BL) and at the end of each intervention phase. Venous blood was collected in the morning prior to the performance test for biochemical measurements and finger blood during the test for glucose determination. Carbohydrate and fat oxidation, energy expenditure, and oxygen use were calculated using respiratory gas analysis.

**Results:**

ALM increased cycling distance during TT by 1.7 km as compared BL (21.9 vs. 20.2 km, P = 0.053) and COK increased 0.6 km (20.8 vs. 20.2 km, P > 0.05). ALM, but not COK, led to higher CHO and lower fat oxidation and less oxygen consumption during TT than BL (P < 0.05), whereas there was no significant difference in heart rate among BL, ALM and COK. ALM maintained higher blood glucose level after TT than COK (P < 0.05). ALM had higher vitamin E and haemoglobin and lower serum free fatty acid (P < 0.05), slightly elevated serum arginine and nitric oxide and plasma insulin (P > 0.05) than BL, and a higher total antioxidant capacity than COK (P < 0.05).

**Conclusions:**

Whole almonds improved cycling distance and the elements related to endurance performance more than isocaloric cookies in trained athletes as some nutrients in almonds may contribute to CHO reservation and utilization and effective oxygen utilization. The results suggest that almonds can be incorporated into diets of those who undertake exercise training for performance improvement.

## Background

Almonds (*Prunus dulcis*) are nutrient dense because they are an excellent source of α-tocopherol, riboflavin, magnesium, and manganese, and a good source of dietary fiber, protein, copper and phosphorus [1,2]. Further, almonds are rich in arginine, a substrate for synthesis of the endothelial dilator, nitric oxide [3]. Almonds are also a source of monounsaturated fats, containing over 9 g per oz (~28 g) [4]. A diverse array of phenolic and polyphenolic compounds, predominantly including flavonoids, e.g., isorhamnetin-3-O-rutinoside and catechin, have been characterized in almonds [5]. This nutrient profile plays an important role in human studies that showed almond consumption was linked to amelioration in biomarkers of oxidative stress [6,7] and inflammation [8,9] and enhancement in LDL resistance against oxidation [10], and improvement in dyslipidemia [11-15]. In July 2003, the U.S. Food and Drug Administration (FDA) approved a qualified health claim stating, “Scientific evidence suggests but does not prove that eating 1.5 ounces per day of most nuts, such as almonds, as part of a diet low in saturated fat and cholesterol may reduce the risk of heart disease.”

Intense, prolonged physical exertion is linked to an increased production of reactive oxygen species (ROS) via oxidative flux into the mitochondrial respiration chain, phagocytic respiratory bursts, and other sources [16]. Such overproduction of ROS overwhelms antioxidant defense capacities, disturbs the balance of immune and endocrine systems, impairs exercise performance, and induces exercise fatigue [17,18]. As almonds are a good source of unsaturated fatty acids, antioxidants and some micronutrients, they may help maintain and/or enhance exercise performance by modulating fuel utilization and strengthening antioxidant defenses. For example, quercetin [19-22] and arginine [23-27] present in almonds may help augment the training effectiveness on exercise performance by up-regulating mitochondrial biogenesis and oxygen sparing capacity and facilitating oxygen delivery to skeletal muscle, and decreasing ammonia liberation.

As of today, the effect of almond consumption on elements of exercise performance in trained athletes remains unknown. We hypothesized that almond consumption could improve exercise performance in trained endurance athletes. The main objective of the study was to investigate whether consumption of almonds would improve elements related to exercise performance as compared to isocaloric cookies in trained athletes participating in annual winter training.

## Methods

### Subjects

Ten trained, male professional athletes (8 cyclists and 2 triathletes) from the same sports team (club) were recruited to participate in the study throughout winter season training in a training camp in the south of China following their training in the north of China. The biometrics of the training subjects are shown in Table 1. Their mean training period was 6.3 ± 1.6 years. They ranked in the top 20 percent of national competition records, and even were champions in Asian games. As professional athletes they trained for 5-6 days a week, and basically participated in national and Asian competitions such as Taiwan/Hong Kong/Hainan/Qinghai Lake bicycle races each year.

**Table 1:** Biometrics of the training subjects

**Biometrics**	**Participants (n = 10)**	**Cyclists (n = 8)**	**Triathletes (n = 2)**
Age (years)	22.3 ± 1.6	23.2 ± 0.8	20.3 ± 0.6
Height (cm)	180.6 ± 7.2	184.0 ± 2.0	172.7 ± 0.6
BM (kg)	74.2 ± 7.7	77.5 ± 2.3	66.5 ± 0.5
VO_2_max (mL/kg/min)	70.3 ± 4.6	70.4 ± 5.6	70.2 ± 0.6
Training years	6.3 ± 1.6	7.2 ± 0.8	4.3 ± 0.6

Key: BM, body mass.

Age (years), height (cm), BM (kg), VO_2_max (mL/kg/min), and Training years (years) for cyclists and triathletes separately and combined.

The study was approved by the Ethical Board of National Institute of Sports Medicine (NISM) and was in compliance with the WMA Declaration of Helsinki. The study protocol was approved by the Review Board of NISM. All athletes signed the consent form before the study.

### Study design, VO_2_max test and food consumption

**Figure 1:**
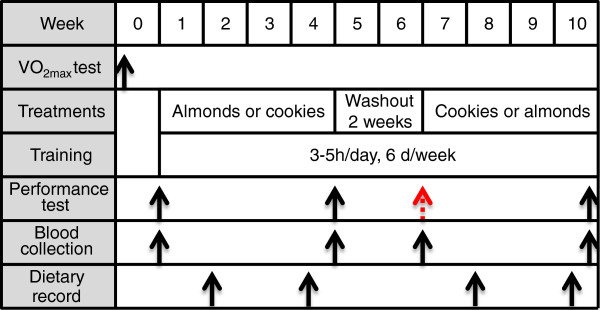
**Study design.** Ten trained male athletes (8 cyclists and 2 triathletes) participated in a 10-week self-controlled, crossover trial during winter season training with training for 3-5 hours per day, 5-6 days a week (see the section of Exercise training regimen and Additional file 4). Dietary treatments consisted of two intervention phases of 75 g raw whole almonds or 90 g isocaloric cookies per day for four weeks each, and a 2-week washout period between two phases. VO_2_max test was undertaken one week prior to the baseline performance test. The time points for performance tests, blood collection and dietary record are indicated with black arrows. The red arrow shows the missed necessary performance test due to modification of athletes’ training plan.

A 10-week self-controlled, crossover design with two 4-week phases of consuming whole almonds and isocaloric cookies in a randomized feeding trial fashion and a 2-week washout period between two phases was conducted (Figure 1). Eight cyclists and two triathletes were randomly assigned to almond- (ALM) and cookies-consuming (COK) groups with equal athlete number after the baseline (BL) performance test.

The individual VO_2_max test was determined using an incremental workload (Watts) on a cycling ergometer (Lode Excalibur Sport, Groningen, Netherlands) 1 week prior to BL performance test. A 100-watt initial workload and 25-watt increment per minute were applied. Respiratory gas was measured using a Cortex MetaMax® 3B remote-controlled system (Cortex Biophysik, Germany), which provides a reliable gas exchange analysis based on the principle of breath-by-breath analysis [28,29]. The system was calibrated with a calibration gas (5% CO_2_, 15% O_2_, BAL. N_2_) (Air Liquide Healthcare America Corporation, Plumsteadville, PA, USA) prior to the VO_2_max test and each performance test.

After the BL performance test, subjects began to consume raw whole almonds (75 g/d as described by Xiao et al. [30]) as 1.8 times the FDA’s claim considered to meet the athletes’ need for intensive training or isocaloric starch-based commercial cookies (90 g/d), which was split equally into three portions fed before three main meals. We chose cookies as the placebo because they are carbohydrate-containing convenient snacks beneficial to exercise training and commonly used by the subjects. Additionally, 90 g of isocaloric cookies have a similar weight but a very different nutritional profile as 75 g of almonds (Additional file 1). In consideration of the unknown effective dosage of almonds for athletes we did not use a lower feeding of almonds. We recognized that the subjects were also aware of almonds as a kind of healthy food, while they seldom had it as snacks due to relative high cost.

Almonds were generously provided by the Almond Board of California. Nutrition information for 75 g almonds and 90 g cookies are presented in Additional file 1.

### Exercise performance test

Subjects stopped their regular training one day before performance test. They reported to testing room either at 8:30 am or 2:30 pm, 1.5 h after standard breakfast or lunch. Each subject used the same indoor stationary bicycle trainer in all 3 performance tests and followed the same testing protocol with the same settings (Additional file 2, a representative video). The same trainer was also used for their routine training. The test consisted of 10 min of warm-up at 30% VO_2_max, 115 min of steady-state (SS) cycling at 50-60% VO_2_max, 20 min of time trial (TT) at all-out effort following a 10-min relaxation (for collection of urine). Expired gas composition and temperature, HR, ambient temperature and humidity during whole TT were monitored using Cortex MetaMax® 3B System and Polar 725 heart rate monitor. Carbohydrate (CHO) and fat utilization was calculated based on the equation built in the software by selecting an assumed 15% total energy expenditure derived from protein.

The rating of perceived exertion (RPE) using the 6-20 Borg scale was surveyed at 20-min intervals throughout the test. The pre- and post-testing body mass (BM) with removal of their racing suit was checked using an electronic BM scale. Urine sample was collected during 10-min relax time of the performance test for volume determination. To ensure subjects were enthusiastic about the test and performed at their highest level, they were informed at the beginning of the test that a prize would be awarded to the winner cycling the longest distance during TT.

### Blood samples collection and biochemical measurements

Venous blood was collected from anticubital arm vein into vacutainer tubes before the performance tests. Heparin plasma and serum were obtained after centrifugation at 3000 × *g* for 10 min. Samples were stored at -80°C until analyses. Finger blood was obtained via puncture for glucose determination at 0, 60, 125 and 155 min during the test.

Free fatty acid (FFA), pyruvic acid (PA), and total antioxidant capacity (TAOC) in plasma were determined using commercial kits (Randox Laboratories Ltd, Crumlin, UK), and an auto-biochemical analyzer (Hitachi, Tokyo, Japan). Plasma VE, malondialdehyde (MDA) and arginine levels, xanthine oxidase (XOD) and glutathione peroxidase (GPx) and superoxide dismutase (SOD) and creatine kinase (CK) activities, and blood urea nitrogen (BUN) and nitric oxide (NO) were measured using spectrophotometric kits (Jiancheng Bioengineering Institute, Nanjing, China). Serum insulin (Ins) and cortisol (Cor) concentrations were measured using radioimmunoassay kit (Jiuding Diagnostic, Tianjin, China). Blood glucose (BG) was determined using handheld blood glucose analyzer (One Touch, LifeScan, Inc. Milpitas, CA).

### Diet and dietary record

All subjects lived in a winter training camp and dined in the same canteen throughout the study, and were advised by a registered dietician to follow a diet with 60% total calories from CHO, 15% from protein, and 25% from fat for 2 days before each performance test. Generally subjects had a typical Chinese breakfast consisting of one chicken egg, two servings of steamed breads or noodles, deep-fried dough sticks, rice congee, bean milk, some meat, some vegetables and appetizers, and lunch and dinner consisting of meat, steamed rice, steamed breads, noodles, soup, milk, fruit and vegetables, etc.

To assess dietary intake throughout the study, a 2-day food record was conducted at week 2, 4, 8, and 10. Chinese food database (issued by Chinese Society of Nutrition) was used for nutritional analysis (Additional file 3). During the regular training, subjects were allowed to drink 6% CHO-electrolytes-vitamins (without VE) beverage (Competitor, Beijing, China) with an average amount of 1500 ml/d. Ten minutes prior to the performance test, subjects checked their BM after emptying bladder, and ingested 2.0% CHO-electrolytes-vitamins (without VE) beverage at 6 mL/kg BM for the pre-testing hydration, 2.5 mL/kg/15 min during SS. No beverage was provided during TT. Subjects did not take any other dietary supplements throughout the study.

### Exercise training regimen

Basically, all subjects had their road cycling training together, whereas two triathletes had their run and swim training in the same training site throughout the study. Briefly, based on their training plan, subjects trained 5-6 days a week with incremental increase in training amount and intensity throughout the study. Detailed content of daily and weekly training was made by coaches on each weekend. The typical daily cycling training regimen consisted of 60-200 km (even 220-250 km) road endurance cycling, 2-3 km*N (N = 2-8) timing sprint cycling on the flat road and sloping fields. Exercise intensity was monitored by HR. Eight cyclists had a weekly road cycling distance of 2840 km and 3110 km during two phases, respectively (Additional file 4). Two triathletes had an average 380-km of road cycling weekly during two phases.

### Limitation of the present study

The original study design included four performance tests performed by subjects before and after each intervention phase during the study. Regretfully, subjects did not undergo VO_2_max test prior to the 2nd intervention phase and the performance test at the beginning of week 7 due to a modified training arrangement. Thus, baseline values of the performance test at the start of the 2nd phase were not available. However, the following 4 points may be helpful to support that the drawback should not affect significance of study outcomes observed at the end of the intervention phases. First, we originally had a crossover design, that is to say, when ALM or COK was compared with BL, there were 5 subjects in each group at the first intervention phase. Second, we had blood biochemistry tests at the end of washout (the end of 6th week). With the exception of a higher FFA, biochemical outcomes after washout at 6th week (MDA 3.7 ± 0.4; XOD 12.5 ± 0.8; TAOC 15.5 ± 1.6; GPx 0.39 ± 0.02; SOD 55.8 ± 0.6; VE 25.2 ± 2.2; CK 237.3 ± 46.4; Cor 19.3 ± 0.8; Hb 143.6 ± 2.7; PA 0.49 ± 0.07; FFA 0.20 ± 0.02; arginine 0.076 ± 0.003; NO 96.7 ± 13.2; Ins 5.0 ± 0.9) were not statistically different from the BL values (see Table 2, their units are the same as shown in Table 2 presented, n = 10). Third, half-life of some nutrients or primarily functional components present in almonds supports that the carry-over effect of the first intervention should be minimal if there was any, e.g, the half-life of α-tocopherol, quercetin, diverse polyphenols and arginine is 57 h [31], 11-25 h [32,33], 1-18 h [34] and 1.5-2.0 h [35], respectively. Finally, subjects all lived and trained in the same training camp throughout the study.

**Table 2:** Blood biochemistries pre-performance tests

**Biomarkers**	**BL**	**COK**	**ALM**
Antioxidant status			
MDA (μmol/L)	3.9 ± 0.15	3.2 ± 0.5	3.2 ± 0.3
XOD (U/L)	13.3 ± 0.4	13.1 ± 0.9	12.4 ± 1.0
TAOC (U/ml)	16.1 ± 0.5	12.8 ± 1.0*	16.3 ± 0.9^#^
GPx (U/ml)	0.41 ± 0.01	0.45 ± 0.05	0.43 ± 0.05
SOD (U/ml)	58.7 ± 1.4	61.2 ± 1.4	59.5 ± 1.4
VE (μmol/L)	19.8 ± 1.8	25.6 ± 1.7	28.7 ± 2.5*
Training, recovery and oxygen-carrying capacity			
CK (U/L)	224.2 ± 32.9	354.7 ± 62.9	288.3 ± 81.1
BUN (mmol/L)	6.5 ± 0.5	7.3 ± 0. 7	6.6 ± 0.6
Hb (g/L)	136.6 ± 2.5	143.2 ± 3.7	145.7 ± 2.7*
Carbohydrate and lipid metabolism production			
BG (mmol/L)	5.6 ± 0.2	5.3 ± 0.3	5.4 ± 0.2
PA (mmol/L)	0.42 ± 0.05	0.44 ± 0.07	0.44 ± 0.07
FFA (mmol/L)	0.22 ± 0.04	0.16 ± 0.03	0.11 ± 0.01*
Metabolism-regulating factors			
Arginine (mmol/L)	0.073 ± 0.005	0.089 ± 0.011	0.113 ± 0.031
NO (μmol/L)	99.6 ± 10.6	113.1 ± 15.3	136.0 ± 18.1
Ins (μIU/ml)	5.5 ± 0.9	5.3 ± 1.6	9.4 ± 2.3
Cor (mmol/L)	20.3 ± 0.9	22.3 ± 2.3	22.0 ± 1.7

MDA, malondialdehyde (μmol/L), XOD, xanthine oxidase (U/L), TAOC, total antioxidant capacity (U/ml), GPx, glutathione peroxidise (U/ml), SOD, superoxide dismutase (U/ml), VE, vitamine E (μmol/L), CK, creatine kinase (U/ml), BUN (blood urea nitrogen (mmol/L), Hb, haemoglubin (g/L), BG, blood glucose (mmol/L), PA, pyruvic acid (mmol/L), FFA, free fatty acid (mmol/L), NO, nitric oxide (μmol/L), Ins, insulin (μIU/ml), Cor, cortisol (mmol/L).

*significantly different from BL at P < 0.05.

^#^significantly different from COK at P < 0.05.

### Statistical analysis

According to the balanced crossover design we combined the data of the same treatment in two phases for statistical analysis.

All results are expressed as mean ± SE except when specified elsewhere. Two-way ANOVA was performed to analyze the differences among groups. Significance was analyzed using post hoc least significant difference (LSD) test. All statistical analyses were performed using SPSS 13.0 software. Differences were considered significant at P < 0.05.

## Results

### Cycling distance

**Figure 2:**
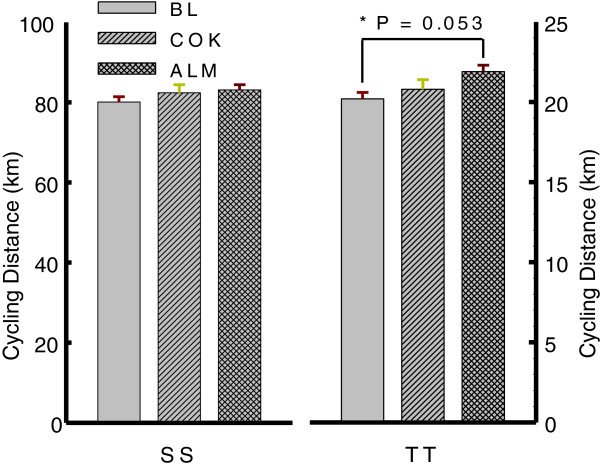
**Cycling distance during TT.** A 20-min time trial at all-out effort was undertaken during TT following a 115-min riding on indoor stationary bicycle trainer at 50%-60% VO_2_max during SS and a 10-min relaxation for urine collection. Cycling distance was recorded by Polar 725 heart rate monitor equipped with a telemeter. ALM (not COK) performed a more cycling distance during TT than BL (*P = 0.053) and COK (P > 0.05). No difference in cycling distance during SS was noted among BL, COK and ALM.

The mean cycling distance during SS phase among BL, ALM and COK was not significantly different (BL, COK and ALM: 80.1 ± 1.3, 82.4 ± 2.0 and 83.1 ± 1.3 km, P > 0.05), while ALM’s distance during TT was 1.7 km (+8.4%) more than BL’s one (21.9 ± 0.4 vs 20.2 ± 0.4 km, P = 0.053), and 1.1 km (+5.3%) longer (21.9 ± 0.4 vs 20.8 ± 0.6 km) than COK (P > 0.05) (Figure 2).

### Rate of perceived exertion

**Figure 3:**
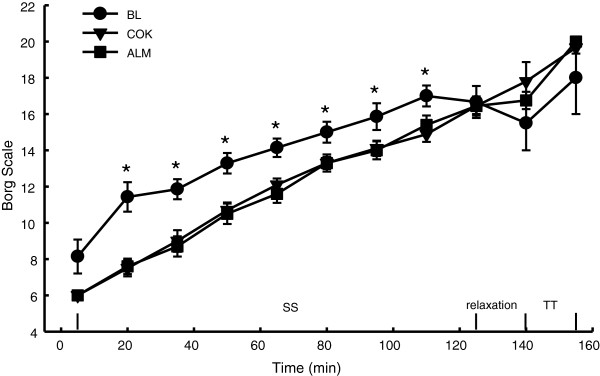
**Time curve of RPE.** RPE (rating of perceived exertion) assessed using a 6-20 Borg scale was recorded every 15 min during performance tests. BL had higher values at some time-points than ALM and COK. No difference between ALM and COK was observed at any time points.

BL showed a higher RPE score at several time points during SS than COK and ALM. No difference among BL, ALM and COK during TT was noted (Figure 3).

### Ambient temperature and humidity, and expired gas temperature

Mean ambient temperature during the performance test at BL was about ~1.3°C higher than COK and ALM (26.9 ± 0.4 vs 25.6 ± 0.3 and 25.6 ± 0.2°C, P < 0.05). The humidity during the performance test at BL was higher than COK and ALM (68.5 ± 1.4 vs 53.2 ± 2.0 and 52.7 ± 1.4%, P < 0.05). Mean expired gas temperature during the performance test at BL was 0.6°C higher than COK and ALM (BL vs COK and ALM: 32.6 ± 0.1 vs 32.0 ± 0.1 and 32.1 ± 0.1°C, P < 0.05).

### BM loss

Mean pre-test BM among BL, COK and ALM was not different. Three groups had a significant BM loss post-test. COK and ALM had a larger magnitude of exercise-induced BM loss post-test than BL (Table 3).

**Table 3:** Change in BM post-performance tests

**Groups**	**Pre-test**	**Post-test**	**BM loss**
	**(kg)**	**(kg)**	**(kg)**
BL	73.9 ± 2.6	72.6 ± 2.6^&^	1.3 ± 0.2
COK	74.7 ± 2.1	72.7 ± 2.1^&^	2.0 ± 0.2*
ALM	74.9 ± 2.4	72.8 ± 2.4^&^	2.1 ± 0.2*

Key: BM, body mass.

^&^significantly different from pre-test in the same group at P < 0.05.

*significantly different from BL at P < 0.05.

### Physiological indicators and gas exchange analysis

**Figure 4:**
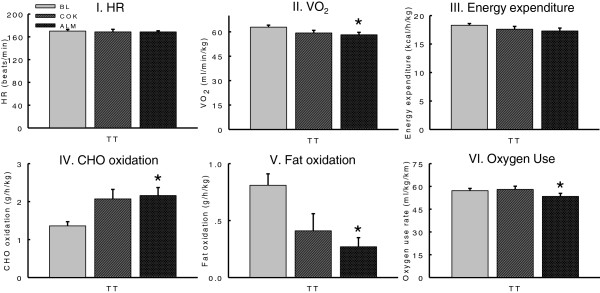
**Main physiological records and gas exchange analysis throughout TT.** Several main physiological parameters (HR, heart rate, and VO_2_, oxygen uptake) throughout TT were recorded as described in the Methods. Energy expenditure (EE), carbohydrate and fat oxidation, and oxygen use were calculated as described in the Methods. No significant differences in HR and EE among BL, ALM and COK (P > 0.05) were found. ALM (not COK) had higher carbohydrate (CHO) oxidation, lower oxygen uptake (VO_2_), fat oxidation and oxygen use as compared with BL (*P < 0.05), whereas there were no difference in VO_2_, CHO and fat oxidation and oxygen use between ALM and COK.

Mean HR, VO_2_, energy expenditure (EE) during TT were not significantly different among BL, COK and ALM. The CHO oxidation during TT in COK and ALM was increased, FAT oxidation and oxygen use rate in both groups was decreased compared with BL. However the change reached a statistical significance only in ALM (Figure 4).

### Blood biochemistries

**Figure 5:**
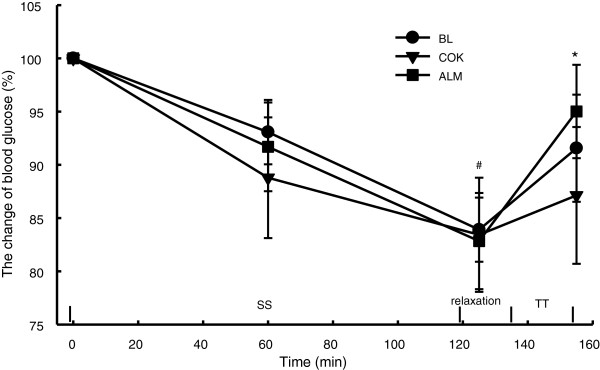
**Change in blood glucose during performance tests.** Blood glucose was tested at 0, 60 min and at the end of SS and TT. The values at the end of SS in BL, ALM and COK were lower than at the start of performance test (^#^P < 0.05). ALM had greater increased percentage at the end of TT than BL and COK as compared to that at the end of SS and a higher level than COK (*P < 0.05) at the end of TT.

Blood glucose was decreased with the progression of SS exercise by ~17% in BL, COK and ALM (P < 0.005). After the 10-min relaxation, blood glucose was increased by 14% and 9% from the end of SS in both BL and COK (P < 0.05), 7% in ALM (P > 0.05). At the end of TT, blood glucose in BL and COK went down as compared with that at the end of SS (P < 0.05), while that in ALM went up (P < 0.05), The difference at the end of TT between ALM and COK tended to be significant (P = 0.054) (Figure 5).

Among the biomarkers reflecting subjects’ antioxidant status, TAOC in COK was decreased, while ALM’s level, which was higher than that in COK, was not changed as compared to BL. ALM, not COK, had a higher blood VE than BL (Table 2). Other indicators were not significantly changed (Table 2).

The indicators of training and recovery, CK and BUN, were not affected by the interventions. Hb in ALM was higher than BL (Table 2).

Serum FFA, but not BG and PA in ALM, which are indicative of carbohydrate and fat metabolic production, were lower than BL (Table 2).

Some metabolism-regulating factors like arginine, NO and Ins, were not different among BL, COK and ALM, whereas ALM had slightly higher levels than COK (Table 2).

### Nutritional intake

The dietary intakes of energy, carbohydrate, total fat (including saturated and mono- and multi-unsaturated fatty acids), protein, total VE and arginine were not different between COK and ALM (Additional file 3).

## Discussion

The present study showed that 4-week consumption of both 75 g/d whole almonds and isocaloric cookies during the winter training season improved cycling distance of time trial and elements of exercise performance relative to BL, with a greater change in the ALM, even though BL’s performance was likely partially affected by relatively high ambient temperature and humidity.

The data suggests that a few notable nutrients/compounds abundant in almonds might improve the effectiveness of the training in a synergistic way via modulating CHO reservation/utilization (by improving glucose transport into skeletal muscle and glycogen synthesis [36,37]), antioxidant capacity [6,7], oxygen transportation/utilization and metabolism regulation [19-26] through slightly raised arginine, insulin, and NO, and statistically increased VE, TAOC and Hb level (Table 2) without greatly affecting fluid balance (Table 3).

In general, training elevates fat-derived energy contribution to an endurance competition [38]. A continuous supply of fatty acids is crucial to athletes participating in distance/endurance competition at moderate intensity, whereas CHO serves as the main fuel during an intense exercise, especially during sprint of a competition [36,39]. Thus, CHO preloading and loading prior to or during a race are essential strategies for athletes participating in an endurance competition [40]. Statistically, our study showed that there were no differences in VO_2_, CHO and fat oxidation during TT between COK and ALM. However ALM had lower VO_2_ and higher CHO oxidation and lower fat oxidation than BL while ALM did not change HR and EE as compared to BL (Figure 3). It should be noted that ALM (not COK) had lower oxygen consumption during TT (Figure 3), lower blood FFA and higher blood glucose at the end of exercise than BL (Figure 5, Table 2), suggesting almonds might help athletes to mobilize more previously reserved CHO, instead of breaking down fat as an energy source during training and the intense exercise [41]. A higher Hb level in ALM might also help athletes transport more oxygen to skeletal muscles during exercise.

L-arginine, the natural precursor of NO, may stimulate insulin secretion [42], decrease oxygen consumption [23,25] and ammonia liberation [27] during exercise and regulate vascular dilation [43,44]. A clinical trial showed that a combined arginine and antioxidant supplement improved exercise performance in the elderly [26]. Insulin facilitates glucose transfer to skeletal muscle tissues and subsequent glycogen synthesis [42,45,46]. Our results suggest that almond consumption may contribute to an improvement in cycling performance- related elements via the effect of arginine on insulin secretion and muscle glycogen synthesis without enhancing insulin sensitivity via down-regulated insulin levels noted in patients with diabetes [14,47,48]. Unsatisfactorily, we did not observe a statistical difference in blood arginine and NO (Table 2) because daily arginine intake from almonds (about 2 g excluding that from the diet) provided ~100 mg/kg BM which was less than that administered in other’s studies [25,27]; athletes had a larger need and utilization (metabolism) of arginine due to intensive exercise; there was a large inter-individual variation; arginine may work with other almond nutrients in a synergistic or additive manner.

Several studies had shown that quercetin alone or plus antioxidants improved mitochondrial biogenesis, VO_2_max, and exercise capacity [19-22]. Therefore, the effect of quercetin on mitochondrial biogenesis and oxygen consumption might also be linked to almond consumption in this study.

Human studies demonstrated that almond consumption increases circulating α-tocopherol concentration in a dose-dependent manner [4,12], decreases biomarkers of oxidative stress in smokers and hypercholesterlemic patients [1,49]. Phenolics in almonds have shown to exert antioxidant action against reactive radicals in humans [6,7]. Thus, a diverse array of phenolic and polyphenolic compounds in almonds might contribute to improving antioxidant capacity in the athletes. Even though ALM (not COK) had a higher blood VE than BL and higher TAOC than COK, we did not find other significant changes related to the antioxidant effects of almond consumption in trained athletes. It is worth noting that the antioxidant effect by almonds was not a predominant factor in improving the elements associated with endurance performance in trained athletes due to their good adaptation to intensive training. Further, one of benefits exerted by almonds might be attributed to decreased inflammation markers (not determined in the study) [8].

## Conclusions

The study showed that almond consumption at 75 g/d for 4 weeks improved time trial distance and the elements related to endurance performance more than did isocaloric cookie consumption in trained Chinese cyclists and triathletes during winter season training when compared to those at the beginning of the training season. Some nutrients/compounds present in almonds like arginine and quercetin might contribute to reserving and using more CHO and enhancing more effective oxygen utilization. Our study suggests that almonds can be incorporated into diets of those who are undertaking exercise training for performance improvement.

## Competing interests

The authors declare that they have no competing interest and that the results of the present study do not constitute endorsement by JISSN.

## Authors’ contributions

MY and LZ were responsible for study design, data collection, statistical analysis, and manuscript preparation. JF, HG, CF, QW, JS, BX, and JL were responsible for biochemical work, dietary record and calculation, data collection/entry, and assistance with manuscript preparation. GH and KL participated in formulating study design. JB and COC helped draft the manuscript. All authors read and approved the final manuscript.

## Supplementary Material

Additional file 1 Nutritional facts of 75 g almonds and isocaloric 90 g cookies. Click here for file

Additional file 2 **A representative video during performance test.** Individual athlete completed three performance tests following the same protocol by riding on the same indoor stationary bicycle trainer using their own training bicycle with the same setting. Click here for file

Additional file 3 Main profiles of dietary nutritional intake for two groups during two phases. Click here for file

Additional file 4 Cyclists’s road cycling training distance during two phases. Click here for file
